# Genetic enhancement of microsomal epoxide hydrolase improves metabolic detoxification but impairs cerebral blood flow regulation

**DOI:** 10.1007/s00204-016-1666-2

**Published:** 2016-02-02

**Authors:** Anne Marowsky, Karen Haenel, Ernesto Bockamp, Rosario Heck, Sibylle Rutishauser, Nandkishor Mule, Diana Kindler, Markus Rudin, Michael Arand

**Affiliations:** 1 Institute of Pharmacology and Toxicology, University of Zurich, Winterthurerstrasse 190, 8057 Zurich, Switzerland; 2 Institute of Complex Systems (ICS-6), Research Center Julich, Wilhelm-Johnen-Straße, 52425 Julich, Germany; 3 Institute of Translational Immunology, University of Mainz, Obere Zahlbacherstrasse 63, 55131 Mainz, Germany; 4 Institute for Biomedical Engineering, ETH Zurich, Wolfgang-Pauli-Strasse 27, 8093 Zurich, Switzerland

**Keywords:** Xenobiotic metabolism, Eicosanoids, Enzymatic mechanism, fMRI

## Abstract

Microsomal epoxide hydrolase (mEH) is a detoxifying enzyme for xenobiotic compounds. Enzymatic activity of mEH can be greatly increased by a point mutation, leading to an E404D amino acid exchange in its catalytic triad. Surprisingly, this variant is not found in any vertebrate species, despite the obvious advantage of accelerated detoxification. We hypothesized that this evolutionary avoidance is due to the fact that the mEH plays a dualistic role in detoxification and control of endogenous vascular signaling molecules. To test this, we generated mEH E404D mice and assessed them for detoxification capacity and vascular dynamics. In liver microsomes from these mice, turnover of the xenobiotic compound phenanthrene-9,10-oxide was four times faster compared to WT liver microsomes, confirming accelerated detoxification. mEH E404D animals also showed faster metabolization of a specific class of endogenous eicosanoids, arachidonic acid-derived epoxyeicosatrienoic acids (EETs) to dihydroxyeicosatrienoic acids (DHETs). Significantly higher DHETs/EETs ratios were found in mEH E404D liver, urine, plasma, brain and cerebral endothelial cells compared to WT controls, suggesting a broad impact of the mEH mutant on endogenous EETs metabolism. Because EETs are strong vasodilators in cerebral vasculature, hemodynamics were assessed in mEH E404D and WT cerebral cortex and hippocampus using cerebral blood volume (CBV)-based functional magnetic resonance imaging (fMRI). Basal CBV_0_ levels were similar between mEH E404D and control mice in both brain areas. But vascular reactivity and vasodilation in response to the vasodilatory drug acetazolamide were reduced in mEH E404D forebrain compared to WT controls by factor 3 and 2.6, respectively. These results demonstrate a critical role for mEH E404D in vasodynamics and suggest that deregulation of endogenous signaling pathways is the undesirable gain of function associated with the E404D variant.

**Electronic supplementary material:**

The online version of this article (doi:10.1007/s00204-016-1666-2) contains supplementary material, which is available to authorized users.

## Introduction

Microsomal epoxide hydrolase is a xenobiotic-metabolizing enzyme, which hydrolyzes potentially genotoxic epoxides to less reactive dihydrodiols. Key features of mEH comprise a broad substrate spectrum and almost ubiquitous expression in all body tissues, with particularly high expression in liver and kidney—consistent with a central role in detoxification (Guengerich 1982; Seidegard and DePierre 1983). mEH substrates comprise epoxides metabolically formed from environmental toxins such as benzene and polycyclic aromatic hydrocarbons (Gonzalez et al. 1982; Jerina 1983; Oesch 1983; Wood et al. 1983) and those from drugs such as phenytoin (Martz et al. 1977) and carbamazepine (Kaneko 1991). In few cases mEH also contributes to the formation of genotoxic metabolites (Bauer et al. 2003; Miyata et al. 1999).

Despite largely unnoticed, mEH is also capable of metabolizing endogenous compounds including epoxy steroids (Fandrich et al. 1995; Vogel-Bindel et al. 1982) and arachidonic acid-derived lipid signaling molecules, so-called epoxyeicosatrienoic acids (EETs) (Marowsky et al. 2009). In a cytochrome P450(CYP)-catalyzed reaction, four EETs regioisomers can be generated, 5,6-, 8,9, 11,12- and 14,15-EETs, which are hydrolyzed by epoxide hydrolases to their respective diols, dihydroxyeicosatrienoic acids (DHETs). DHETs are presumably less biologically active or exert different biological activity than their parent molecules (Froemel et al. 2012; Spector 2009). EETs have been implicated in a variety of physiological functions, ranging from vasodilation (Campbell and Fleming 2010), angiogenesis (Webler et al. 2008; Yang et al. 2009), cell proliferation (Panigrahy et al. 2013) and inflammation (Node et al. 1999) to pain (Spector 2009; Terashvili et al. 2008; Wagner et al. 2011). In brain, EETs can directly dilate cerebral arteries (Amruthesh et al. 1992; Ellis et al. 1991) and contribute importantly to neurovascular control (Iliff et al. 2009; Peng et al. 2002). EETs-generating cells in the CNS comprise neurons (Qu et al. 2001), astrocytes (Alkayed et al. 1996), and endothelial cells (Medhora et al. 2001). While astrocyte-derived EETs are thought to play a pivotal role in neurovascular control, endothelium-derived EETs seem to contribute to the endo-dependent modulation of vasomotor tone by agonists such as bradykinin (Gebremedhin et al. 1998). The role of mEH in endogenous lipid metabolism is largely neglected, because the bulk of EETs are metabolized by the more rapid sister enzyme, soluble epoxide hydrolase (sEH). Although mEH displays higher affinity for EETs compared to sEH, in particularly for the 8,9- and 11,12-regioisomer, sEH clearly outperforms mEH in terms of maximal velocity (*V*_max_) (Marowsky et al. 2009).

**Fig. 1:**
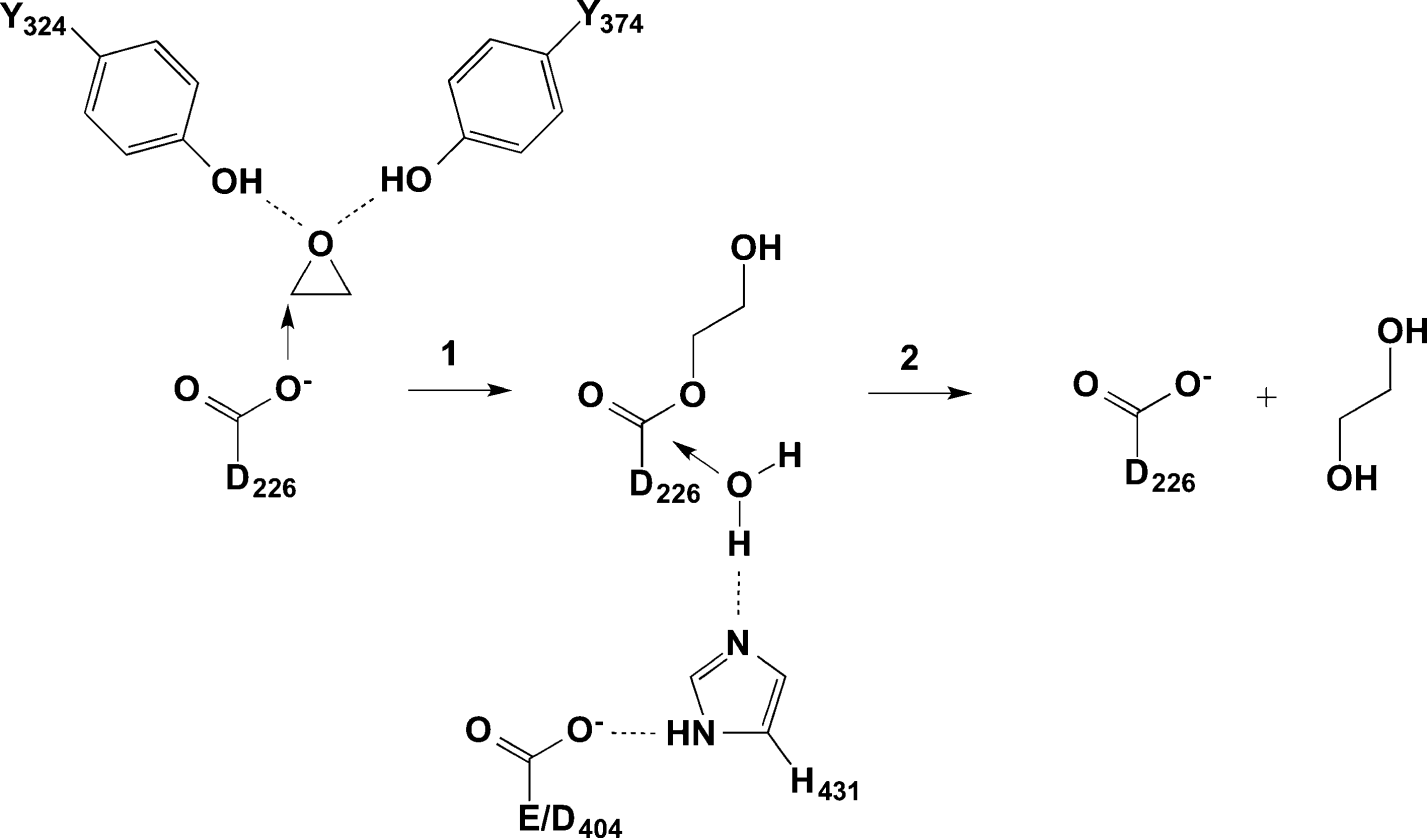
The two-step enzymatic mechanism of microsomal epoxide hydrolase. In the first step, mEH forms a covalent bond to its substrate (for the sake of simplicity, only the epoxide core of the substrate is depicted in the scheme). The resulting ester is subsequently hydrolyzed in step 2. Note that step 2 is rate limiting and that it is substantially accelerated by changing E404 to D

*V*_max_ of mEH can be substantially modulated by specific amino acid exchanges. A dramatic increase in mEH activity is caused by an amino acid replacement in the catalytic triad (Fig. 1) of the enzyme (Arand et al. 1999). While in most species including humans and all other vertebrates, mEH proteins contain a glutamic acid at the position equivalent to amino acid 404 in the human enzyme, a few taxonomically lower species, in particular several insects and some molds of the *Aspergillus* genus, carry an aspartic acid at this site. When introduced into the rat mEH protein, this amino acid exchange Glu404Asp (mEH E404D) showed a 23-fold and 39-fold enhancement in *V*_max_ for the substrates styrene-7,8-oxide and 9,10-epoxystearic acid, respectively (Arand et al. 1999). The amino acid at position 404 forms a charge relay system together with H431, which activates water through proton abstraction, with the mutation E404D quickening this second, rate-limiting step of the enzymatic reaction (Arand et al. 1999) (Fig. 1).

In humans, two functional polymorphisms in the EPHX1 gene in exon 3 and 4 have been shown to alter enzyme activity, probably due to enhanced stabilization of the enzyme rather than a substantial change in enzyme kinetics as in mEH E404D (Hassett et al. 1994a, b; Omiecinski et al. 2000). Specifically, a decrease in enzymatic mEH activity, linked mostly to the H113 allele, was reported to increase the risk for several types of cancer such as colon, ovarian, lung and liver cancer (Erkisi et al. 2010; Goode et al. 2011; Harrison et al. 1999; Lee et al. 2011; Zhong et al. 2013a, b). By contrast, the high activity genotype has been related to pre-eclampsia, a life-threatening hypertension affecting pregnant women (Groten et al. 2014; Pinarbasi et al. 2007; Zusterzeel et al. 2001). This disease is almost exclusive to humans and characterized by abnormal maternal uterine vascular remodeling (Pennington et al. 2012).

The absence of the mEH E404D variant in higher animals suggests that in humans an elevated detoxification rate would come with a high price. We hypothesized that mEH is critically involved in the metabolism of endogenous compounds such as vasoactive EETs and that an increased activity level of the enzyme would negatively affect EETs-controlled vascular processes. To test this notion, we generated mice harboring the rapid mEH E404D variant and assessed them for detoxification efficiency and physiological aberrations.

## Materials and methods

See supplemental informations.

## Results

### Generation and characterization of mEH E404D mice

For the generation of the mEH E404D mice, a single point mutation was introduced into the last exon of the mEH gene of murine embryonic stem cells via homologous recombination that resulted in the desired amino acid exchange E404D in the enzyme protein. From these genetically modified stem cells, a mouse line was generated on a genetic C57BL/6 background (for details on the construction see Supplemental Information, Fig. S1).

**Fig. 2:**
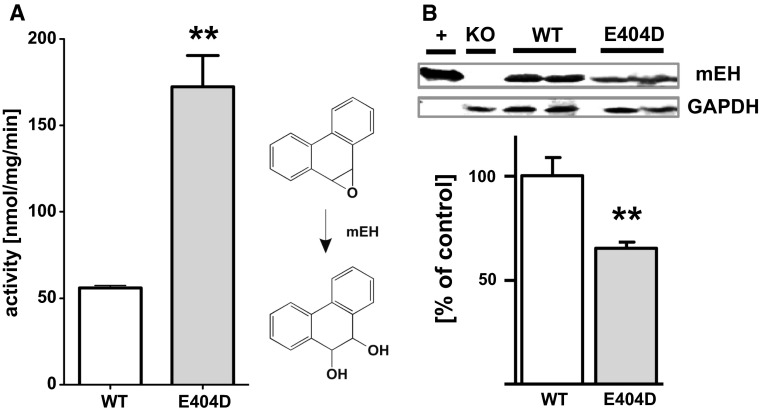
Enhanced detoxification capacity of the mEH E404D mutant. **a** Liver microsomes from mEH E404D detoxifies phenanthrene-9,10-oxide significantly faster than microsomes from WT. Preparations from both genotypes (*n* = 3, each) were incubated with the genotoxic substrate, and turnover was analyzed spectrophotometrically as described under “Materials and methods” section (see Supplementary Information). Results are presented as mean ± SD. **b** mEH E404D protein levels are reduced to 65 ± 4 % of mEH WT protein levels in mouse liver, as demonstrated by ratiometric comparison with GAPDH expression in immunoblot analysis. Purified recombinant mouse mEH (+) and microsomes from mEH (−/−) mice (KO) served as the positive and the negative control for the specificity of the immune detection. Precise quantification was achieved using an Odyssey^®^ infrared fluorescence imager. Results are expressed as the mean (*n* = 4 per genotype) ± SEM. **P < 0.01

The mEH E404D mice displayed no obvious differences in size, weight, development and general behavior compared to WT littermates, thus exhibiting no overt phenotype. In particular, litter sizes were indistinguishable between mEH E404D × mEH E404D and WT × WT control breeding pairs, arguing against the occurrence of a spontaneous preeclampsia-like pathology in female pregnant mEH E404D mice (Ahmed et al. 2010). To assess the detoxification efficacy of the mutant, liver microsomes from mEH E404D and WT animals were incubated with phenanthrene-9,10-oxide, a genotoxic, mEH-selective substrate. Enzymatic activity of mEH E404D microsomes was increased threefold compared to WT microsomes (Fig. 2a), demonstrating that the mEH variant displayed in fact significantly higher turnover. To rule out that increased mEH expression levels underlie the observed acceleration in turnover, hepatic mEH protein levels were quantified. Immunoblotting using an anti-mEH antibody revealed a significant downregulation of mEH E404D protein content to 65 % of mEH protein level found in WT liver, indicating strong compensatory adaptations (Fig. 2b) and an actually 4.5-fold faster detoxification of the mutant as compared to the WT enzyme under our assay conditions. In kidney, mEH E404D protein was also significantly downregulated to 68 % of the corresponding WT level, but no such changes in expression levels were noted in lung and cerebral cortex from mEH E404D compared to WT control animals (data not shown).

### Contribution of mEH E404D to arachidonic acid metabolism in liver

**Fig. 3:**
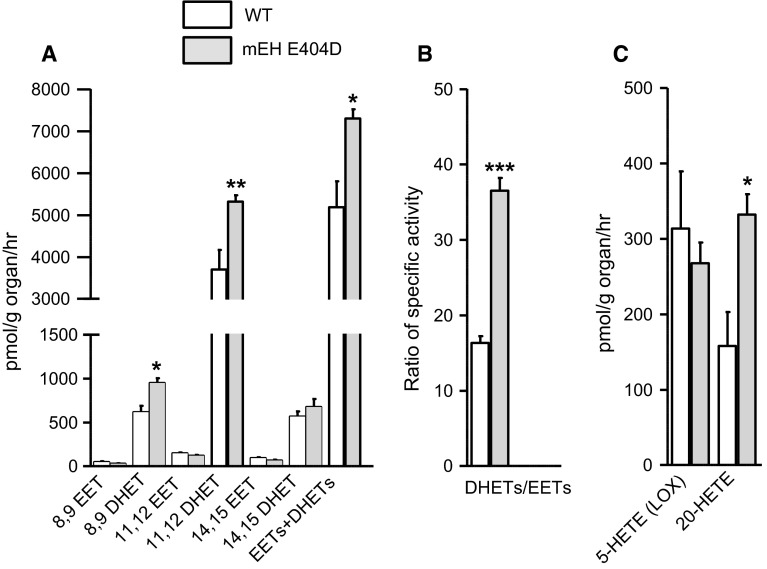
mEH E404D liver tissue homogenate shows substantially higher EETs turnover compared to WT control despite significant downregulation of mEH E404D protein. **a** Incubation with 30 μM arachidonic acid leads to similar EETs, but significantly higher DHETs levels in mEH E404D liver compared to WT control (*n* = 5 per genotype). Consequently, the sum of [EETs + DHETs] is higher in mEH E404D, pointing toward an increase in epoxygenase activity. **b** A significantly higher DHETs/EETs ratio in mEH E404D liver compared to WT indicates accelerated turnover by the mEH variant. **c** The CYP product 20-HETE, a strong vasoconstrictor, is generated in higher amounts in mEH E404D compared to control. 5-HETE, a product of the LOX pathway, is generated in similar amounts in both genotypes (*n* = 5 per genotype). Results are presented as mean ± SEM. *P < 0.05; **P < 0.01; ***P < 0.001

Liver is also crucial for the metabolism of endogenous compounds, including fatty acids and EETs (Sacerdoti et al. 2003). To assess the impact of mEH E404D on arachidonic acid metabolism, mEH E404D and WT liver homogenates were incubated with arachidonic acid (AA; 30 µM, 60 min) and metabolites were determined via LC–MS/MS. Compared to WT, the mEH variant showed clearly enhanced DHET levels for the 8,9- and 11,12-regioisomers, while total EETs levels remained similar across genotypes (Fig. 3a, Supplementary Tab. S1). Consequently, the DHETs/EETs ratio was significantly enhanced in mEH E404D liver compared to WT (Fig. 3b). sEH activity was the same in both genotypes, as evidenced by 14,15-EET turnover rates of 60.5 ± 8.8 nmol/mg/min in mEH E404D liver cytosol and of 65.8 ± 10.5 nmol/mg/min in WT liver cytosols (*n* = 5 per genotype; *P* = 0.412). Therefore, the accelerated turnover rate of EETs was due to the enhanced mEH activity—despite of the marked downregulation of mEH E404D protein (Fig. 2b). Furthermore, the sum of total EET and DHET levels was higher in mEH E404D liver relative to WT (Fig. 3a), indicative of increased CYP activity in the mEH variant. Likewise, levels of the CYP4A/4F-product, 20-HETE, were also increased in mEH E404D liver, whereas levels of 5-HETE, a product of the competing lipoxygenase pathway (LOX pathway), remained unchanged (Fig. 3c). Morphology and histology of mEH E404D liver were normal (data not shown). Taken together, mEH E404D liver showed enhanced EETs turnover, a specific increase in CYP epoxygenase and CYP ω-hydroxylase activity and unchanged activity for the 5-LOX pathway and sEH-mediated EET hydrolysis relative to WT controls.

Analysis of EETs and DHET levels in plasma and urine also revealed consistently higher DHETs/EETs ratios in mEH E404D compared to WT samples, primarily due to increased turnover of 8,9- and 11,12-EETs (see Supplementary Tab. S1), demonstrating a systemic rather than a liver-specific effect on EETs metabolism by the mEH variant.

### Comparison of enzymatic properties between purified mEH WT, sEH and mEH E404 protein

Comparison of EETs/DHETs ratios between mEH E404D and WT controls for liver, plasma and urine pointed toward an E404D-specific regioisomer profile. Indeed, when mEH E404D and WT liver microsomes were incubated with a mixture of 8,9-, 11,12- and 14,15 EETs (10 µM each) in the presence of the sEH inhibitor tAUCB (10 μM), mEH E404D showed highest turnover rates with 8,9-EETs, followed by 11,12 > 14,15 EETs (Table 1). This is different to mEH WT, which shows similar rates for the 8,9- and 11,12-regioisomer. For a more detailed analysis of the enzymatic profile of mEH E404D, we compared the activity of purified murine sEH, mEH WT and mEH E404D protein with the different EETs regioisomers as substrates. These turnover assays revealed that purified sEH clearly outperforms mEH WT with regard to maximal velocity (*V*_max_) and catalytic efficiency (*k*_cat_/Km), irrespective of the regioisomer (Table 1). However, the E404D point mutation boosted the catalytic efficacy of the WT enzyme fivefold for 8,9-EETs and threefold for 11,12-EETs, respectively, with the result, that purified sEH and mEH E404D were equally efficient in metabolizing 8,9-EETs.

**Table 1:** Regiopreference and kinetic constants of WT and mEH E404D with EET regioisomers

	Turnover^a^ (%)		Vmax (nmol/mg/min)			Km (µM)			kcat/Km (M^−1^ × s^−1^) × 10^3^		
	WT	E404D	WT	E404D	sEH	WT	E404D	sEH	WT	E404D	sEH
8,9-EET	52 ± 6	88 ± 1**	120	3000	2400	0.85	4.5	2	120	600	500
11,12-EET	61 ± 6	78 ± 1*	67	4300	11,000	0.22	4.5	0.6	275	860	5000
14,15-EET	18 ± 1	34 ± 4*	20	260	20,000	0.18	3.4	2	100	68	5000

^a^Turnover of a mixture of EET regioisomers (10 μM each) with equal amounts of either WT or E404D mEH liver microsomes in the presence of the sEH inhibitor tAUCB

*p ≤ 0.05, **p ≤ 0.01, Student’s *t*-test

### Role of mEH E404D in acetazolamide-induced vasodilation in cerebral cortex and hippocampus

**Fig. 4:**
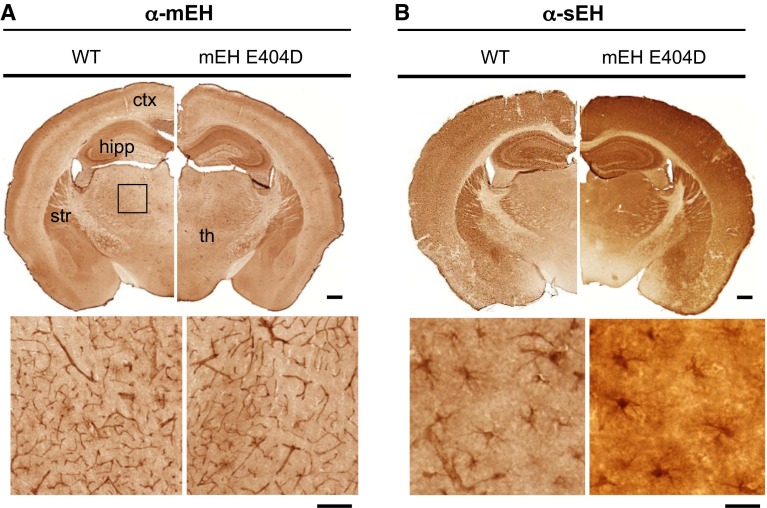
Expression pattern of mEH and sEH is unchanged in mEH E404D compared to WT brain. Distribution of mEH and sEH illustrated by immunoperoxidase staining. **a** Comparison of mEH distribution on transverse sections from WT (*left*) and mEH E404D (*right*) animals, showing strong mEH IR in the hippocampal formation, striatum and outer layers of the cerebral cortex (*upper row*). Endothelial cells throughout the slice are mEH positive. The area of the rectangle is magnified in the *lower row*, showing thalamic vessels. **b** Comparison of sEH distribution in transverse slices from WT (*left*) and mEH E404D (*right*) animals in comparison. sEH immunoreactivity is mainly found in astrocytes throughout the brain. The area in the rectangle in the cortex is magnified, showing astrocytes with their star-like protrusions. *Scale bar upper row* 0.5 mm. *Scale bar lower row* (**a**) = 100 µm, (**b**) = 20 µm. *ctx* cortex, *hipp* hippocampus, *str* striatum, *th* thalamus

**Fig. 5:**
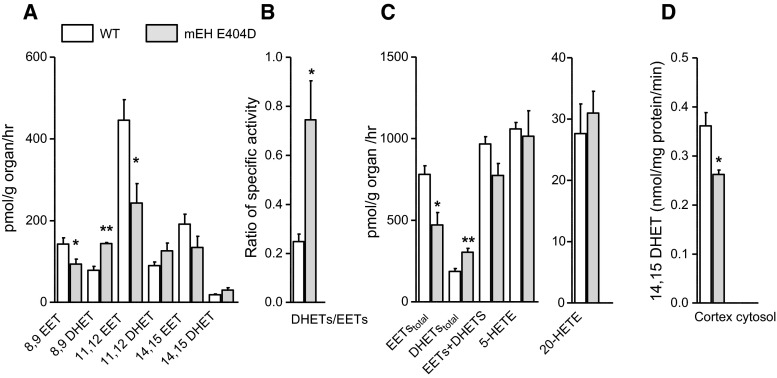
Cortical tissue from mEH E404D mice displays lower EETs and higher DHET levels relative to its WT counterpart. **a** Incubation with 30 μM AA leads to a genotype-specific EETs-DHETs profile with pronounced differences for the 8,9- and 11,12-regioisomer (*n* = 5 for each genotype). **b** DHETs/EETs ratios are higher in mEH E404D cortex compared to WT, reflecting the accelerated EETs turnover by the mEH variant. **c** Total [EETs + DHETs] levels, 20-HETE and 5-HETE levels did not differ between genotypes, indicative of unchanged activity of the CYP and LOX pathway in mEH E404D cortex. **d** Turnover of 14,15-EETs (10 μM) to 14,15 DHETs by mEH E404D and WT cortex cytosol revealed reduced sEH activity in mEH E404D cortex (*n* = 5 for each genotype). Results are presented as mean ± SEM. *P < 0.05; **P < 0.01

Despite an increase in EETs turnover by mEH E440D in liver, urine and plasma, we did not observe any gross physiological aberrations. Because phenotypes often manifest themselves only after a challenge, we searched for a paradigm where the observed alterations in enzyme characteristics should have an impact. EETs are strong vasodilators in cerebral blood vessels, and mEH is expressed in cerebral vascular and perivascular structures (Marowsky et al. 2009). Therefore, we focused our investigations on cerebral hemodynamics. Brain mEH and sEH expression did not differ between mEH E404D and WT, and strong mEH immunoreactivity (IR) was detected throughout cerebral endothelial cells, a subpopulation of cortical astrocytes and in specific neuronal subtypes (Fig. 4). sEH IR was found in astrocytes with particularly strong expression in the forebrain. We chose cortex and hippocampus as regions of interest (ROIs), because they show similar sEH, but different mEH expression. Specifically, in hippocampus mEH is found in principal neurons (CA1-CA3 pyramidal cells), whereas in cortex mEH-positive neurons are absent. To study the effect of the mEH mutant on AA metabolism in brain, cortex and hippocampal tissue homogenates from mEH E404D and control animals were incubated with AA (30 μM, 60 min). Similar to liver, DHETs levels and DHETs/EETs ratios were elevated by factor 1.3 and 2.9 in mEH E404D hippocampus and cortex, respectively, compared to WT (Fig. 5a−c, Supplementary Tab. S1). Concentrations of 20-HETE, an essential vasoconstrictor, were similar across genotypes (Fig. 5c, Supplementary Tab. S1). sEH activity was reduced in E404D compared to WT cortex samples, indicated by lower hydrolysis of 14,15-EETs in cortical cytosol (Fig. 5d), implying that higher turnover is primarily due to mEH E404D activity.

Results obtained in tissue homogenates have the disadvantage that EET- and DHET-producing cells are not identified. As endothelial cells express CYP epoxygenases, generating EETs, as well as mEH, we studied their metabolic profile in more detail. Cerebral endothelial cells were isolated from total WT and mEH E404D brains and EETs/DHETs levels analyzed. EETs-to-DHET levels were substantially shifted with similar total [EET + DHET] levels, suggesting similar CYP epoxygenase activity, but 3.6 times higher DHET/EET ratio in mEH E404D compared to WT cells (Supplementary Tab. S1).

**Fig. 6:**
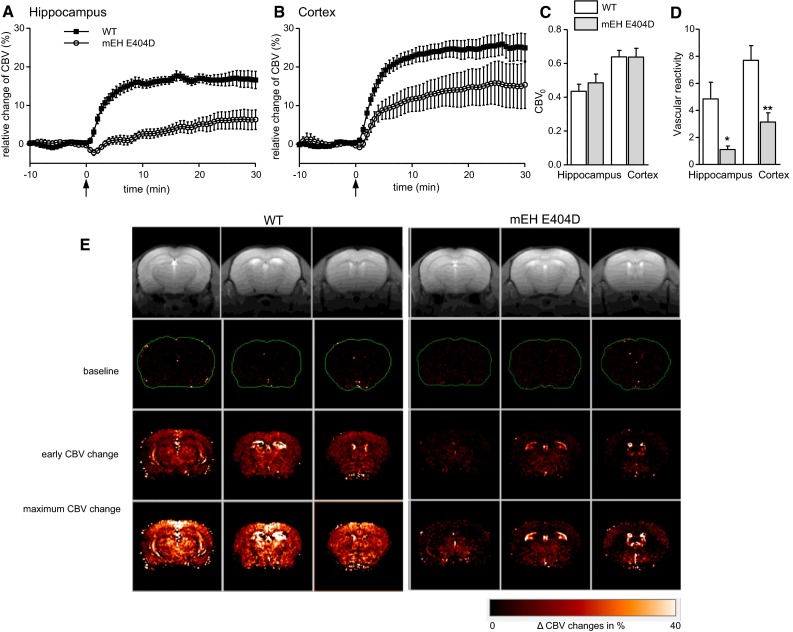
Acetazolamide injection leads to significantly lower CBV responses in two frontal brain areas of mEH E404D animals compared to WT controls. Relative changes of CBV of mEH E404D (*open symbols*) and WT control (*filled symbols*) in the hippocampal (**a**) and somatosensory (ss) cortical (**b**) ROI measured by fMRI. Black arrows indicate i.v. infusion of AZ. WT animals show ΔCBV_%_ of comparable magnitude in both brain areas, compared to which those of mEH E404D animals are distinctly lower. Comparison over the entire CBV intensity curve after AZ injection (0–30 min) reveals a highly genotype-specific difference for both brain areas (repeated-measures ANOVA, ss cortex *p* = 0.0027, hippocampus *p* < 10–6). Data are presented as mean ± SEM. **c** Absolute baseline CBV values (CBV0) measured by fMRI vary with brain region, but are similar between genotypes. **d** Vascular reactivity is severely compromised in mEH E404D brains compared to WT controls. **e** *Color-coded* CBV maps with representative images for WT controls (*left*) and mEH E404D (*right*) brains illustrate the genotype-specific response to AZ injection. Anatomical MR reference images are shown in the *top row*. *Color-coded* CBV maps superimposed on the anatomical scans represent (from *upper* to *lower row*) baseline ΔCBV_%_ values, early changes in ΔCBV_%_ (3 min post-AZ injection) and maximum ΔCBV_%_ values (13 min post-AZ injection). For each genotype rows from left to right correspond to Bregma −2.92, −2.06 and +0.62, respectively. Hippocampus is visible in the first and second row, ss cortex in the third row for each genotype. *P < 0.05; **P < 0.01

To test whether the altered EETs metabolism in mEH E404D has a functional impact, we studied the vasodilatory response in mEH E404D and WT brain to a pharmacological stimulus, the carbonic anhydrase inhibitor acetazolamide (AZ). Quantitative analysis of fMRI signals was carried out in the somatosensory cortical and hippocampal ROIs, for which AA metabolism rate had been determined before. Prior to AZ administration (i.v.), basal CBV_0_ were analyzed for a genotype-specific difference. CBV_0_, which reflects vascular architecture and tonus, did not differ between mEH genotypes in both brain areas (Fig. 6c). Following AZ injection, we observed a rapid increase in relative CBV values in WT somatosensory cortex and hippocampus, reaching a stable plateau after 15 min with ΔCBV_%_ values of 24.9 ± 0.5 % in somatosensory cortex and 16.8 ± 0.3 % in hippocampus (average intensity in the time interval 15–30 min after AZ injection). By contrast, the CBV response in the mEH mutant was markedly decreased with ΔCBV_%_ values of 11.1 ± 0.7 and 4.8 ± 0.4 % for cortex and hippocampus, respectively (Fig. 6a, b). Statistical analysis of ΔCBV _%_ versus time profile in response to AZ administration revealed a significant genotype-specific difference for both brain areas. Functional maps reflected the results derived from the intensity profiles. Here, early CBV changes (ΔCBV_%_ at 3 min) comprise the initial slope as a measurement for vascular reactivity, while maximum CBV changes (ΔCBV_%_ at 13 min) reflect the maximum vessel dilatation in response to AZ (Fig. 6e). Quantitative analysis of the vascular reactivity [initial slope, (dΔCBV_%_/d*t*)_*t*=0_] yielded significantly lower values for mEH E404D animals compared to WT in both brain areas (Fig. 6d). Furthermore, the functional maps illustrated that CBV changes in the somatosensory cortex were representative for the entire cortex in both genotypes. To exclude the possibility that the reduced CBV-fMRI response in mEH E404D animals was due to a reduced sensitivity of the pharmacologic target of the AZ challenge, namely the carbonic anhydrase, transcutaneous p_tc_CO_2_ values were measured in drug-naïve WT and mEH E404D animals (Princz-Kranz et al. 2010). Injection of AZ led to a significant augmentation of p_tc_CO_2_ in both genotypes and no genotype-specific difference in CO_2_ levels could be detected (see Supplementary Tab. S2). Taken together, the AZ-induced hemodynamic response in cortex and hippocampus of mEH E404D brain was severely compromised compared to WT, evidenced by significantly lower values for ΔCBV_%_ and vascular reactivity.

## Discussion

In the present paper, we demonstrated that enhancement of mEH activity significantly improves hepatic detoxification, but at the same time shifts the ratio DHET:EET to higher values in vivo due to an enhanced turnover of EETs. As a probable consequence of this imbalance, cerebral vasodilation is strongly attenuated after AZ injection relative to WT. Gross pathological changes on morphological and histological levels were not observed in mEH E404D mice, but several strong tissue-specific adaptations in enzyme levels occurred, which presumably counteracted or at least diminished the effects of the powerful mEH E404D. In liver (and kidney, data not shown), mEH E404D protein levels were decreased by one-third. Hepatic CYP epoxygenase activity was upregulated, such that EETs levels were unchanged in mEH E404D relative to WT, and only DHET levels differed between genotypes. Finally, cortical sEH activity was substantially reduced in mEH E404D animals.

We used AZ as pharmacological stimulus and fMRI-based analysis as technique-of-choice, because it allows for simultaneous analysis of CBV changes throughout the brain, including deeper cortical layers and hippocampus. In both brain areas, AZ-induced increase in CBV was blunted in mEH E404D compared to control animals. AZ has a well-documented effect on cerebral hemodynamics and is used in clinical settings to evaluate cerebrovascular reactivity and reserve in patients with occlusive cerebrovascular diseases at risk for cerebral ischemia (Imaizumi et al. 2004; Vagal et al. 2009). Mechanistically, AZ acts by selectively inhibiting carbonic anhydrase and induction of hypercapnia (Taki et al. 1993), which leads to an increase in cerebral blood flow and CBV (Frankel et al. 1992; Zhou et al. 2009). On the molecular level, an involvement of EETs in AZ-induced CBV change seems likely. Specifically, the molecular targets identified in AZ action overlap with those reported for EETs. Both EETs and protons, the latter generated by AZ-induced acidosis, were shown to activate K_ATP_ channels (Wang et al. 2003). Furthermore, EETs and protons both modulate BK channel activity via ryanodine receptor-modulated calcium waves in vascular smooth muscle cells (Dabertrand et al. 2012; Earley et al. 2005; Knot et al. 1998). In our study the AZ-induced CBV changes correlate well with the DHET/EETs ratios in E404D and WT brain tissue without any indication for a genotype-specific difference in the hypercapnic effect. This strongly suggests a downstream cross-talk between these signaling pathways.

mEH has long been regarded as mainly, if not exclusively, xenobiotic-metabolizing enzyme. This notion was based on the fact that its sister enzyme sEH, in purified form and under optimized conditions, is orders of magnitude faster than mEH in the turnover of fatty acid epoxides (Chacos et al. 1983). In contrast, our present findings strongly suggest a prominent role of mEH E404D in control of EET levels and thus of EET-dependent signaling pathways, exemplarily shown in this study for the cerebral hemodynamics.

The question remains if mEH-mediated EETs turnover only becomes significant after genetic acceleration or if already the WT enzyme plays a significant role in EET metabolism. There are several arguments supporting the latter concept. First, although *V*_max_ of WT mEH is by orders of magnitudes lower than that of sEH, its catalytic efficacy, the physiologically relevant parameter, is, with 0.2 × 10^6^ per molar and second, in a range compatible with physiological turnover of EETs. Second, the *K*_M_ of the WT enzyme with EETs is very low (sub-micromolar), indicating a high (apparent) affinity of EETs as mEH substrates. Third, mEH has a kinetic advantage over sEH due to its (sub)cellular localization: mEH and CYP epoxygenases are co-expressed in endothelial cells at least throughout the murine brain and in a subpopulation of cortical astrocytes. Furthermore, both are ER-resident with their catalytic domains facing toward the cytosol (Holler et al. 1997). Such physical proximity might favor direct interaction or “substrate channeling” from CYP epoxygenases to mEH, thus bypassing the cytosol-residing sEH. In this scenario the latter might only come into play, once the enzymatic capacity of mEH is saturated. mEH WT and mEH E404D differ distinctly in this parameter with saturation levels shifted to higher substrate concentrations by one order of magnitude in the accelerated variant relative to WT (Arand et al. 1999).

The hepatocyte is one cell type that simultaneously displays high expression levels of all three entities, i.e. AA-epoxygenating CYPs, mEH and sEH. Hepatocytes constitute more than 90 % of the liver mass and thus dominate our AA turnover experiments with liver homogenates. sEH expression in mouse liver is substantially higher than that of mEH and so is its catalytic efficacy (see Table 1) with all three EET regioisomers. Nevertheless, a relatively moderate enhancement of the mEH catalytic efficacy produced a significant shift in the DHET-to-EETs -ratio for the 11,12-regioisomers. This demonstrates that the close proximity of mEH to epoxygenases in conjunction with its high affinity for EETs is clearly able to compensate for its comparatively lower catalytic efficacy (for a more extensive discussion including quantitative aspects see Supplementary Information).

The EPHX1 gene can be found throughout almost all kingdoms of live (Cavalier-Smith 2010), including bacteriae, protozoae, chromistae, fungi and animals, with the interesting exception of plants. A dedicated search for E404D-like variants throughout all living organisms reveals its actual presence in a small percentage of species, in particular in a few bacteria, insects, nematodes and in some molds of the *Aspergillus* genus, but, so far, a complete absence in the around 200 vertebrate species for which EPHX1 sequence data have been deposited (M. Arand, unpublished observation). If present in insects and molds, this apparently goes along with at least one second EPHX1 gene in the given species that harbors a glutamic acid residue in the charge relay system [see, for example, multiple mEHs in the red flour beetle (Tsubota et al. 2010)]. This strongly suggests that higher species—with the exception of plants—depend on the presence of the glutamic acid variant of mEH with its—in terms of *V*_max_—restricted turnover rate, most likely to allow a controlled fine tuning of epoxide-related signaling molecules. Finally, the common human EPHX1 polymorphisms indicate a potential involvement of mEH in the regulation of vascular tone: distinct human EPHX1 polymorphisms associated with slightly enhanced enzymatic activity predispose its carrier to pre-eclampsia, a pregnancy-related pathology with hypertension as a leading symptom (Groten et al. 2014; Pinarbasi et al. 2007; Zusterzeel et al. 2001).

An obvious question that remains is why we do not have the fast mEH404D variant in lower amounts? On first sight, this seems much more economical. Yet one needs to keep in mind that only the second step of catalysis is faster with the mEH E404D, while the first step, the formation of the enzyme-substrate ester, is as fast as in the WT enzyme. This first step already detoxifies reactive substrates of the enzyme. In the liver, where the bulk of xenobiotic metabolism takes place, the high expression level of mEH creates the unusual situation of this enzyme often being in excess over its substrates. This allows for the efficient detoxification by just forming the metabolic intermediate with the substrate without the need of immediate hydrolysis. Less enzyme, even when regenerated much faster as would be the case with the mEH E404D mutant, would result in higher steady-state concentrations of toxic epoxides, based on the law of mass action (Arand et al. 2003) rather than in more efficient detoxification.

## Electronic supplementary material

Below is the link to the electronic supplementary material.

Supplementary material 1 (PDF 905 kb)
