# Supplementary material for: Genetic enhancement of microsomal epoxide hydrolase improves metabolic detoxification but impairs cerebral blood flow regulation
Source: Arch Toxicol. 2016 Feb 2;90(12):3017–27. doi: 10.1007/s00204-016-1666-2 (PMC5104800; doi:10.1007/s00204-016-1666-2)
Supplement: Supplementary file 1 — Supplementary material 1 (PDF 905 kb) [file 204_2016_1666_MOESM1_ESM.pdf]

Supplementary Information

Materials and Methods

Construction of mEH E404D knock in mice.

An mEH gene fragment was isolated from a 129 SVJ mouse genomic library in the bacteriophage lambda FIX II (Stratagene, La Jolla). In brief, 1 x 10^6 plaque forming units from the library were divided into thirty pools and amplified via infection of E. coli strain XL-1 blue MRA (P2) (Stratagene, La Jolla) in liquid culture. PCR analysis of the DNA isolated from these cultures using the mEH intron 4-specific primers 5'-AAGCCCTTGCTGATGGTGC-3' (forward primer) and 5'-ACTTCTGGAAGCCCAGCCG-3' (revers primer) identified two pools that contained mEH genomic DNA, as indicated by a 1.4 kb mEH-specific amplicon. From one of these pools, a phage clone was isolated via plaque lifting (Sambrook et al. 1989) that contained an 18 kb mouse genomic insert representing the 3'-end of the mEH gene, including exons 4 - 9, plus approximately 8 kb downstream region. Restriction analysis and successful PCR amplification of all expected exons proved the authenticity of the clone that was subsequently used to construct the targeting vector. Multiple cloning steps led to a final construct (see Fig. S1A), harbouring a mouse genomic fragment that extends from positions 180986300 to 180997697 of chromosome 1, based on ensemble genome assembly GRCm38 (http://www.ncbi.nlm.nih.gov/projects/genome/assembly/grc/mouse/). This gene fragment was engineered to contain a single G => T point mutation in position 180989935, leading to the desired amino acid substitution E to D in codon 404 of the mature mEH protein and, at the same time, to the generation of a new, diagnostically useful EcoRV restriction site. Furthermore, the gene fragment was interrupted at position 180988008, 1.55 kbp downstream of the polyadenylation site of the gene, by a neomycin phosphotransferase expression cassette with the same transcriptional orientation as the gene itself, to allow for the selection of recombination positive clones. In addition, the gene fragment was flanked by thymidine kinase expression cassettes on both sides to improve the ratio of homologous recombinants over random integrants on negative selection. This construct was transfected in 129 ES cells and homologous recombinants were identified by Southern blot analysis of clones obtained via double selection with G418 and ganciclovir (see Fig. S1A for the location of the outer (probe 1) and inner (probe 2) hybridization probe, and Fig. S1B for a representative Southern blot analysis). Positive clones were used for blastocyst injection and subsequent implantation into pseudopregnant mice. Presence of the recombinant allele was analyzed by combined PCR amplification/EcoRV restriction, using the oligonucleotides 5'-CCCTGGAAGATCTGCTGACTAACA-'3' and 5'-CTTCGAAGGCAGCAAAGTGG-'3' as forward and revers primers. The

resulting 673 bp amplicon was reduced in size to 576 bp by EcoRV digestion in case of the mutant allele and remained unaltered for the WT allele (see Fig. S1C for a representative analysis, where samples 1,2,6 and 7 represent heterozygotes, samples 3 – 5 are from homozygous knock in animals and sample 8 is from a WT mouse). Positive individuals were used as founders and were crossed back on a C57/BL6 background for 10 generations. The resulting line was designated C57/BL6 mEH-E404D KI, or short mEH KI.

Western Blot Analysis for mEH protein quantification

Pre-weighed liver samples, 4 of each genotype, were sonicated on ice in 10 vol RIPA buffer (20mM Tris, 150mM NaCl, 1% Triton-X100, 1% Sodium deoxycholate, 0.1% SDS, protease inhibitor), centrifuged at 10´000xg and the supernatant was collected. Protein concentrations were determined using Bradford protein assay. Aliquots were run on 12.5% tricine sodium dodecyl sulfate polyacrylamide gel, transferred to polyvinylidene fluoride membrane and immunoblotted with rabbit anti-rat mEH antibody (1:5000 made in house) and monoclonal anti-mouse GAPDH (1:4000, Sigma G8795) followed by infrared-fluorescent-labeled secondary antibodies (donkey anti-rabbit IgG, 800CW; donkey anti-mouse IgG, 700CW, IRDye, Li-Cor Biosciences, USA). Detection of infrared fluorescence in the 700 and 800 nm channels was carried out with an Odyssey IR imager (Li-cor Biosciences, USA), followed by densitometric quantification with Odyssey IR imager software.

Expression of mouse mEH WT, mEH E404D and sEH protein and EETs turnover assay

Expression and purification of recombinant mouse mEH, mEH E404D and sEH were carried out as described in detail in (Marowsky et al. 2009). For the expression of mEH E404D, the respective point mutation was introduced into the pET20 mEH construct with the Quikchange procedure (Stratagene) using the mutation primers 5’- CAG CCT TCC CTT CTG AT*A* TCC TGC ATG CCC CA-3’ and 5’- GGG GCA TGC AGG AT*A* TCA GAA GGG AAG GCT GA-3’ (the italicized letter indicates the point mutation and the underlined sequence represents the resulting EcoRV recognition site). The recombinant proteins were purified via metal affinity chromatography using a HisTrap column (AP Biotech), followed by size exclusion chromatography. The purified proteins (0. 2–100 ng) were incubated with 8,9-, 11,12- and 14,15-EETs (0.1–32 µM) in Tris buffer (10 mM, pH 8.0), containing NaCl (100 mM), EDTA (1mM) and gelatin (0.1% w/v) for 10 min at 37 °C in a final volume of 50 µl. Reaction was terminated after 10 min by addition of an equal volume of acetonitrile. After centrifugation for 3 min at 13’000xg in a benchtop centrifuge, the supernatant was analyzed by LC-MS/MS as described below.

Quantification of oxylipids in tissue samples

Standard-protocols were used with minor modifications. Organs (liver, cerebral cortex, hippocampi) were frozen in liquid nitrogen and pottered in 1 ml PBS buffer, pH 6.8. Peroxide-free arachidonic acid in ethanol (90010.1, Cayman Chemical) was added in presence of a NADPH-regenerating system and samples were incubated at 37°C for one hour. Reaction was stopped by heating the samples to 95°C for 5 min, put on ice and 500µl acetonitrile/methanol (1:1) including deuterated standards (8,9-EET-d_{11}, 11,12-EET-d_{11}, 14,15-EET-d_{11}, 14,15-DHET-d_{11}, 14,15-EET-$d_{11,}$ 5-HETE-$d_{8,}$ 20-HETE-$_{d6}$) were added. After incubation at -80°C for 30min, samples were sonified and centrifuged for 5min at 13000xg in a benchtop centrifuge. The supernatant was diluted with 4ml ice-cold H_2O, containing 0.1% formic acid. Oxylipids were extracted using Isolute ® C18 solid phase extraction columns (Biotage, Uppsala; 100 mg sorbent mass). Plasma oxylipids were isolated from 100 to 200 µl mouse blood treated with 1/10 volume of 3.8% sodium citrate. After centrifugation for 20min at 2500xg, plasma was diluted with one volume of methanol/acetonitrile (1:1) including deuterated internal standards (same as above). Samples were centrifuged for 5min at 13000xg and the supernatant was diluted with 8 vol water. Formic acid was added to a final concentration of 0.1%. Oxylipids were extracted using solid phase extraction as described above.

For oxylipid detection in urine, animals were kept in metabolic cages for 24 hours and total urine was collected in presence of the antioxidant butylated hydroxytoluene (10mg, MP Biomedicals). 500µl methanol/acetonitrile (1:1) including deuterated standards were added to 250µl of the 24-hour urine and kept at -80°C overnight. The following day, samples were sonicated and 200µl 10 M NaOH was added. After incubation at 60°C for 30min, 400µl 100% acetic acid were added and the samples were centrifuged (5min, 13'000xg). The supernatant was diluted with 3.65 ml water and passed onto solid phase extraction columns as described above.

Preparation of liver and cortex microsomes and cytosol

100-200 mg frozen tissue from WT and mEH E404D animals was homogenized on ice in 1ml buffer containing 0.125M KCl, 0.25M sucrose, 0.1M potassium phosphate, 1mM EDTA, pH 7.4. The samples were centrifuged in two steps (5min at 600xg; 20min at 9'000xg). The supernatant was taken and centrifuged for 30min at 100´000xg in a TLA 100.3 rotor using an Optima® benchtop ultracentrifuge (Beckman), which yielded the cytosol as supernatant. The pellet, containing the microsomes, was dissolved in 500µl resuspension buffer (0.125M KCl and 0.1M Tris (pH 7.4)) and finally shock-frozen in liquid nitrogen.

mEH activity assay with phentanthrene-9,10-oxide
Spectrophotometric recordings of the mEH-specific turnover of phenanthrene-9,10-oxide was essentially carried out as described by Armstrong and colleagues (Armstrong et al. 1980), adapted to a microplate reader format. In brief, 0.5 µg liver microsomal protein was added to a 10 µM solution of phenanthrene-9,10-oxide in Tris-HCl, 10 mM, EDTA, 1 mM, NaCl, 100 mM, pH 8.0, to a final volume of 300 µl in a polystyrene microtiter plate well, mixed well, and the decrease in OD_{290} was monitored for 30 min in 15s intervals at 25 °C in a Spectramax 250 microplate reader (Molecular Devices). Although the baseline absorption at 290 nm was already substantial, due to the absorption by the plate itself, sensitive monitoring of phenanthrene-9,10-oxide hydrolysis was still possible. Under these conditions, a $\Delta_{\varepsilon} = 2400\ M^{-1}$ was used to calculate the turnover rate, based on an approximate d = 0.57 cm in the microtiter well.

sEH activity assay with 14,15-EETs
Cytosol samples were diluted in 50µl 10mM Tris buffer (100mM NaCl,1mM EDTA, pH 7.4) and 14,15-EET in ethanol (Cayman Chemicals) was added to a final concentration of 10 µM. Samples were incubated for 10 min at 37°C and reaction was stopped by adding 50 µl acetonitrile containing 10 µM sEH inhibitor tAUCB (a kind gift from Christophe Morisseau, UC Davis, CA). 100 µl water containing deuterated standards were added and the samples were centrifuged at 13'000 rpm for 10 min (4°C). The supernatant was immediately subjected to LC-MS/MS.

LC-MS/MS analysis of oxylipids
Separation of analytes was performed on a Gemini C18 NX column (2 x 150 mm, 5 µm pore size) with a corresponding opti-gard pre-column using an Agilent 1100 liquid chromatography system. The mobile phase consisted of freshly prepared (A) water containing 0.0125% ammonia and (B) acetonitrile containing 0.0125% ammonia at a flow rate of 350 µl/min using an injection volume of 40 µl. Compounds were eluted using a linear gradient from 16 to 26 % buffer B for 2 min, followed by a linear gradient from 26 to 36% buffer B within 16 min and from 36 to 95% within 0.7 min. An isocratic flow of 95% B was held for 1.6 min and finally the column was re-equilibrated for 4.2 min with 16% B. The HPLC system was coupled to a 4000 QTRAP hybrid quadrupole linear ion trap mass spectrometer (Applied Biosystems) equipped with a TurboV source and electrospray (ESI) interface. Analytes were recorded using multiple reaction monitoring in negative mode (-MRM) using the following source specific parameters: IS -3800V, temperature 550°C, curtain gas (CUR = 30), nebulizer gas (GS1 = 50), heater gas (GS2 = 50) and collision gas (CAD = medium). The compound specific parameters for the different substrates were determined by direct infusion of standard solutions

(1-3 ng/ml) in methanol at a flow rate of 10µl/min using the quantitative optimization function of the Analyst software 1.5.2. Samples were quantified by determining the peak AUC with the Multiquant software using the transitions specified in Tab. S3. Values were corrected for the extraction efficiency using the internal standard peak area. The limit of detection (LOD) ranged from 0.0002 - 0.002 pmol/mg tissue and the limit of quantification from 0.0005 to 0.005 pmol/mg tissue, corresponding to a signal-to-noise ratio of 3 and 10, respectively.

CBV measurements by fMRI

A detailed description of the method is given in (Princz-Kranz et al. 2010). In short, experiments were performed on a Pharmascan MR system 47/16 (Bruker BioSpin, Germany) operating at a magnetic field strength of 4.7T. The functional image protocol consisted of a RARE sequence with the following parameters: temporal resolution = 40s, TR=3333.33ms, TE=8.12ms, RARE factor =32, FOV=2.00 x 1.34 cm, MD=133x100, SLTH=1.00mm, ISD=1.5mm,, N_{slices}=8 and NA=4. The corresponding voxel dimension were 150 x 134 x 1000 μm^3. For the actual experiment, mice were anesthetized with an initial dose of 3% isoflurane given i.v., which was reduced to 1% for fMRI measurement, and the head was fixed in a stereotaxic fixation. A single dose of 21 mg/kg of the neuromuscular blocking agent gallamine (Sigma Aldrich) was administered i.v.. The experiment consisted of three parts: 1. Eight sequential images were recorded yielding the baseline signal intensity S_{pre} followed by the injection of a bolus of Endorem® (Guerbet SA, Roissy, France) at a dose of 60 mg Fe/kg. 2. After 20 min allowing the contrast agent concentration to reach a steady state, 30 sequential baseline images were acquired yielding the signal intensity S_0. 3. At image 31, acetazolamide (AZ) (30 mg/kg; Goldshield Pharmaceuticals Ltd, Croydon, UK) was administered i.v. as a slow bolus. CBV values were computed on a voxel-by-voxel basis and image analysis was carried out using Biomap (Novartis Institute for Biomedical Research, Basel, Switzerland). Regions of interest (ROIs) were defined on the appropriate image slice using the mouse atlas by Paxinos et al.

Transcutaneous measurements of pCO_2 ($p_{tc}CO_2$) values

$p_{tc}CO_2$ measurements are proportional to arterial pCO_2 values (Ramos-Cabrer et al. 2005), with the advantage that for transcutaneous measurements no blood samples need to be taken. Measurements were performed using a transcutaneously operating blood gas analyzing device TCM4 (Radiometer Medical ApS, Denmark). The measuring electrode was stuck to the shaved back of the animal, lateral of the spine. A 20-min time window was used for equilibration, during which isoflurane, applied i.v., was reduced from 3 to 1%. Baseline $p_{tc}CO_2$ values were recorded every 2 seconds for 20 min followed by injection of 30 mg/kg AZ. Recordings were continued for another 40 min, corresponding to the duration of the fMRI experiment.

Statistics

Unless otherwise stated unpaired Student's *t* test were used to compare single parameters between genotypes. One-way ANOVA, followed by Newman-Keuls post hoc test and ANOVA repeated measurements were used when appropriate (SPSS software and GraphPad Prism5).

Discussion

Quantitative considerations on the contribution of mEH to 11,12-EET turn-over in mouse liver homogenates.

The following quantitative estimates are based on two plausible assumptions:

1) In an uncompartmented model, the contribution of a given enzyme to the turnover of a substrate under non-saturating conditions should be proportional to its abundance multiplied by its catalytic efficacy.

2) Saturation of a high affinity enzyme should reduce its overall contribution in a mixture of enzymes when others remain unsaturated and can still increase in turnover rate with increasing amounts of substrate.

If these two conditions are accepted, the contribution of mEH to 11,12-EET turnover in liver homogenates can be estimated using the abundance and catalytic efficacy of mEH and sEH. In our laboratory, we find the mEH to contribute around 0.6 % to the microsomal protein content and sEH around 0.8 % to the cytosolic protein content in C57/BL6 mouse liver, well in agreement with data reported in the literature (Knowles and Burchell 1977; Meijer and Depierre 1985). In addition, varying amounts of sEH are found in microsomes, due to the co-sedimentation of sEH-containing peroxisomes with ER vesicles during microsome preparation, which is not taken into account in the following calculation but would, at best, decrease the overall mEH contribution to substrate turnover. In C57/BL6 liver, the cytosolic protein content is approximately fourfold that of the microsomal protein content (Hammock and Ota 1983). Taken together, there is about 5 times higher amounts of sEH in C57/BL6 liver as compared to WT mEH. At the same time, we find the catalytic efficacy of sEH to be about 20-fold higher with 11,12-EET, compared to WT mEH (5000:275 = 18.2). This leads to the conclusion that, if liver was an uncompartmented system, sEH should contribute 100-fold (5 x 20-fold) more to 11,12-EET turnover than mEH or, in other words, mEH contribution to 11,12-EET turnover should be around 1 %. On the introduction of the E404D mutation, we change both, mEH abundance (reduced to 65%) as well as catalytic efficacy (increased by a factor of 3), which collectively increases the contribution according to the above assumption by a factor of two, thus to 2 % overall contribution. Due to biologic variation and analytic imprecision, such a contribution would almost certainly escape detection. Nevertheless, we find that introducing the E404D mutation substantially

increases the 11,12-EET turnover rate as evidenced by a significantly increased 11,12-DHET/EET ratio (WT: 16.3; mEH E404D: 36.5), despite an apparently increased 11,12-EET formation rate (11,12-EET + DHET in WT: 5.2 nmol/g liver/h; 11,12-EET + DHET in mEH E404D: 7.3 nmol/g liver/h). This clearly demonstrates a substantial contribution of mEH to EET turnover in mouse liver homogenates, even in the presence of high amounts of sEH within the same preparation, which is best explained by its close vicinity to the EET-forming CYPs.

References

Armstrong RN, Levin W, Jerina DM (1980) Hepatic microsomal epoxide hydrolase. Mechanistic studies of the hydration of K-region arene oxides. J Biol Chem 255(10):4698-705

Hammock BD, Ota K (1983) Differential induction of cytosolic epoxide hydrolase, microsomal epoxide hydrolase, and glutathione S-transferase activities. Toxicol Appl Pharmacol 71(2):254-65

Knowles RG, Burchell B (1977) Mouse liver epoxide hydratase: purification and identity with the rat liver enzyme [proceedings]. Biochem Soc Trans 5(3):731-2

Marowsky A, Burgener J, Falck JR, Fritschy JM, Arand M (2009) Distribution of soluble and microsomal epoxide hydrolase in the mouse brain and its contribution to cerebral epoxyeicosatrienoic acid metabolism. Neuroscience 163(2):646-61 doi:10.1016/j.neuroscience.2009.06.033

Meijer J, Depierre JW (1985) Properties of cytosolic epoxide hydrolase purified from the liver of untreated and clofibrate-treated mice. Purification procedure and physiochemical characterization of the pure enzymes. Eur J Biochem 148(3):421-30

Princz-Kranz FL, Mueggler T, Knobloch M, Nitsch RM, Rudin M (2010) Vascular response to acetazolamide decreases as a function of age in the arcA beta mouse model of cerebral amyloidosis. Neurobiol Dis 40(1):284-92 doi:10.1016/j.nbd.2010.06.002

Ramos-Cabrer P, Weber R, Wiedermann D, Hoehn M (2005) Continuous noninvasive monitoring of transcutaneous blood gases for a stable and persistent BOLD contrast in fMRI studies in the rat. NMR Biomed 18(7):440-6 doi:10.1002/nbm.978

Sambrook J, Fritsch EE, Maniatis T (1989) Molecular Cloning - A Laboratory Manual, vol 1-3, 2nd edn. Cold Spring Harbor Laboratory Press

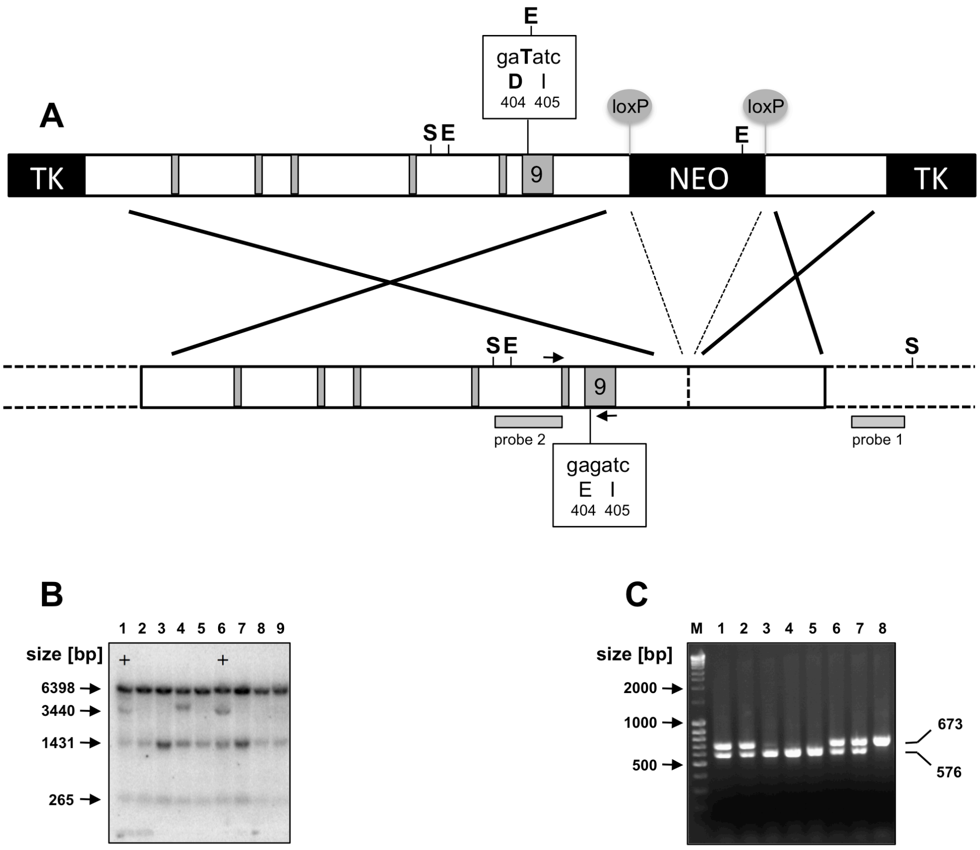

Fig. S1 Targeting construct composition and genotypic analysis. **A** Graphical representation of the construct composition (upper bar) and the desired homologous recombination event with the respective genomic region (lower bar). The large arm of the syngenic sequence within the construct contains the 3' end of the mEH gene, including the exons 4 – 9 (grey boxes). The point mutation to be introduced is located in the terminal exon 9 and is depicted in the text boxes above/below the targeting construct/the genomic sequence. As evident, exchanging G against T results in the desired alteration of the encoded protein sequence and the simultaneous generation of a novel EcoRV restriction site. The syngenic sequence is interrupted by a neomycin phosphoribosyltranferase expression cassette (NEO, shown as black box) inserted in a native EcoRI restriction site 1.55 kb downstream of the polyadenylation site of the mEH gene. The resulting small arm of the targeting construct represents the 1.71 kb genomic sequence downstream of the EcoRI site. The total syngenic region is flanked by thymidine kinase expression cassettes (TK, shown as black boxes) on either side. Not shown here is the pBR322-based plasmid backbone of the construct. Restriction sites (S = Sst I, E = EcoRV) and the location of hybridization sites for P^{32}-labelled probes (grey bars, probe 1 = external probe, probe 2 = internal probe) used for the screening of recombinant ES cells, as well as the hybridization sites for PCR primers used for genotyping of animals (black arrows) are also shown. **B** Representative Southern blot analysis of transfected ES cell clones after combined G418/ganciclovir selection. Genomic DNA was

isolated from the ES cells, restricted with SstI and EcoRV and blotted on a PVDF membrane after electrophoretic separation on an agarose gel. A mixture od P^{32}-labelled probes 1 and 2 was used to simultaneously check for homologous recombination (probe 1) and insertion of the desired point mutation (probe 2). The 6398 bp fragment observed with all clones 1-9 is the large EcoRV/SstI fragment recognized by probe 1 in the unmodified wild type DNA, which is present at least in one copy, given the diploid nature of ES cells. The 3440 bp fragment also hybridizes with probe 1 and is indicative of the homologous recombination event, due to the insertion of the new EcoRV restriction site situated in the NEO expression cassette. The 1431 bp fragment recognized by probe 2 is the EcoRV/EcoRV fragment specific for the targeting construct and proves the presence of the point mutation, but does not discriminate between homologous recombination and random insertion. The 265 bpEcoRV/SstI fragment is recognized by probe 2 and does not discriminate between wild type and recombinant allel. An occasionally observed 5234 bp fragment (not shown here) is the EcoRV/EcoRV probe 2-detected fragment resulting from a homologous recombination event that incorporated the NEO cassette correctly but did not modify the desired locus in exon 9. In the present analysis, clones 1 and 6 display the desired restriction/hybridization pattern while clones 2,3 5,7,8 and 9 lack the fragment of 3440 bp, which is best explained by random integration of the targeting construct. The pattern of clone 4 is not explained by either random integration or regular homologous recombination. **C** Restriction analysis of a genomic PCR fragment for rapid genotyping of mEH knock in mice. Primers located in exons 8 and 9 were used to amplify a 673 bp genomic fragment from ear biopsies of transgenic mice to prove the presence of the point mutation via subsequent release of a respective 576 bp fragment by EcoRV restriction, followed by agarose gel analysis. In the present representative example, samples 3,4 and 5 indicate homozygous knock in mice, samples 1, 2, 6 and 7 represent heterozygous individuals and sample 8 shows a WT animal.

Tab. S1 EET and DHET concentrations in plasma, urine, liver and brain, as well as brain endothelial cells of mEH E404D and WT mice

	EETs		DHETs		Ratio DHET/EETs	
	WT	mEH E404D	WT	mEH E404D	WT	mEH E404D
Native content						
Plasma [pmol/ml] (3)	22.8 ± 2.2	10.9 ± 2.1*	15.2 ± 1.1	11.6 ± 1.9	0.67 ± 0.04	1.07 ± 0.07**
Endothelial cells [pmol/200'000 cells] (3)	0.66 ± 0.13	0.48 ± 0.01	0.24 ± 0.04	0.65 ± 0.04**	0.38 ± 0.04	1.37 ± 0.10***
Alkaline lysis						
Urine [pmol/24 hours] (3)	6.9 ± 1.6	11.5 ± 0.9	2.1 ± 0.3	4.6 ± 0.5*	0.30 ± 0.01	0.44 ± 0.16
Arachidonic acid metabolism rate						
Liver [pmol/g organ/hour] (3-5)	288 ± 19	216 ± 19*	4886 ± 555	7105 ± 168*	16.3 ± 0.8	36.5 ± 1.33***
Cortex [pmol/g organ/hour] (3-5)	780 ± 54	471 ± 77*	187 ± 16	304 ± 24**	0.25 ± 0.03	0.74 ± 0.16*
Hippocampus [pmol/g organ/hour] (3-5)	1103 ± 62	1148 ± 146	496 ± 15	663 ± 50*	0.46 ± 0.03	0.59 ± 0.03*

	Ratio 8,9 DHET/EETs		Ratio 11,12 DHET/EETs		Ratio 14,15 DHET/EETs	
	WT	mEH E404D	WT	mEH E404D	WT	mEH E404D
Native content						
Plasma (3)	1.67 ± 0.27	2.65 ± 0.16*	0.32 ± 0.02	0.60 ± 0.06*	1.13 ± 0.09	1.46 ± 0.26
Endothelial cells (3)	n.d.	n.d.	0.37 ± 0.06	2.02 ± 0.58*	0.39 ± 0.03	1.10 ± 0.14**
Alkaline lysis						
Urine (3)	0.52 ± 0.16	0.77 ± 0.01	0.10 ± 0.01	0.18 ± 0.01**	0.61 ± 0.07	0.80 ± 0.11
Arachidonic acid metabolism rate						
Liver (3-5)	13.50 ± 2.60	28.80 ± 4.00*	24.40 ± 1.70	50.10 ± 3.40**	6.20 ± 0.60	12.10 ± 2.10*
Cortex (3-5)	0.56 ± 0.08	1.71 ± 0.22**	0.22 ± 0.04	0.66 ± 0.19	0.11 ± 0.03	0.26 ± 0.06
Hippocampus (3-5)	0.62 ± 0.04	0.95 ± 0.08*	0.95 ± 0.07	1.08 ± 0.07	0.41 ± 0.02	0.49 ± 0.03

Values represent means ± SEM. The number of biological samples analyzed per mean are given in brackets after the specification of the respective sample. Differences between genotypes have been statistically evaluated by unpaired, two-sided Student's t-test and significance is indicated by stars appended to the mEH E404D values.
*p<0.05; **p<0.01;***p<0.001

Tab. S2 pCO_2 response to acetazolamide treatment in WT and E404D mice

	Baseline	t=0	t=10 min	t=20min	$\Delta p_{tc}CO_2$ (peak - baseline)
**WT (9)**	35.3 ± 3.2	36.1 ± 3.1	61.1 ± 2.9	63.4 ± 3.3	26.9 ± 2.7
**mEH E404D (9)**	39.3 ± 3.5	41.1 ± 3.5	64.2 ± 3.9	67.9 ± 4.3	26.7 ± 2.1

pCO_2 values are given in millimeter of mercury (mm Hg). Statistical analysis (one-way ANOVA) revealed no significant difference between genotypes with regard to average baseline, absolute $p_{tc}CO_2$ values at timepoints t=0, t=10, t=20 and with regard to the difference between baseline and peak.

Tab. S3 Technical parameters for oxylipin detection by LC-MS/MS

Compound	RT [min]	Transition [m/z]	Function	CE [V]	CXP [V]	EP [V]	DP [V]
8,9-EET	11.0	319.2 → 68.9	Quantifier	-30	-1	-10	-70
		319.2 → 301.1	Qualifierf	-16	-7	-10	-70
11,12-EET	10.3	319.2 → 167.1	Quantifier	-18	-13	-10	-65
		319.2 → 301.1	Qualifier	-16	-7	-10	-70
14,15-EET	9.3	319.2 → 301.1	Quantifier	-16	-7	-10	-70
		319.2 → 218.8	Qualifier	-16	-13	-10	-70
8,9-DHET	7.5	337.2 → 126.8	Quantifier	-28	-7	-10	-70
		337.2 →123.0	Qualifier	-28	-1	-10	-70
11,12-DHET	6.8	337.2 → 166.8	Quantifier	-26	-11	-10	-70
		337.2 → 168.9	Qualifier	-26	-11	-10	-70
14,15-DHET	6.4	337.2 → 206.9	Quantifier	-26	-15	-10	-70
		337.2 → 128.8	Qualifier	-26	-9	-10	-70
5-HETE	11.3	319.2 → 114.8	Quantifier	-24	-5	-10	-80
		319.2 → 301.1	Qualifier	-18	-13	-10	-80
20-HETE	7.4	319.1 → 275.1	Quantifier	-24	-15	-10	-100
		319.1 → 301.2	Qualifier	-24	-3	-10	-100

RT, Retention time; CE, collision energy; CXP, collision exit potential; EP, entrance potential; DP, declustering potential
